# An Event-Related Potentials Study on the Syntactic Transfer Effect of Late Language Learners

**DOI:** 10.3389/fpsyg.2021.777225

**Published:** 2022-01-03

**Authors:** Taiping Deng, Dongping Deng, Qing Feng

**Affiliations:** ^1^School of Marxism Studies, Zhejiang University of Finance and Economics, Hangzhou, China; ^2^No. 3 Middle School of Gan County, Ganzhou, China; ^3^College of Education, Hebei Normal University, Shijiazhuang, China

**Keywords:** transfer effect, input training, syntactic representation, P600, malleability

## Abstract

This study explored the syntactic transfer effect of the non-local subject-verb agreement structure with plural head noun after two intensive phases of input training with event-related potentials (ERP). The non-local subject-verb agreement stimuli with the plural head nouns, which never appeared in training phases, were used for the stimuli. A total of 26 late L1-Chinese L2-English learners, who began to learn English after a critical period and participated in our previous experiments, were asked back to take part in this syntactic transfer experiment. Results indicated that a significant ERP component P600 occurred in the key region (the verb) of the sentences with syntactic violations in the experimental group, but none occurred in the control group. This demonstrated that there was a significant transfer effect of the input training. The possible theoretical explanation was provided and also the malleability of the late L2 learners was discussed.

## Introduction

### The Input Factor and L1 Representation Entrenchment

In the L1 field of syntactic acquisition, cognitive grammar theory such as the usage-based models advocates that input is an important factor in representation entrenchment. Each time the structure (input) is encountered, the representation could be entrenched deeper (Barlow and Kemmer, 2000; Langacker, 2000; Bybee, 2006).

Studies on the differences in L1 speakers provide evidence for the role of input in representation entrenchment (Dabrowska, 1997, 2008a,b; Kayne, 2000; Chipere, 2001; Luka and Barsalou, 2005; Brooks and Sekerina, 2006; Street and Dąbrowska, 2010; Luka and Choi, 2012). For example, Chipere (2001) tested two groups of L1 English adult students from the same school: low academic attainment students (LAA in abbreviation) with a score “D” or below in English curricula and a high academic attainment group (HAA in abbreviation) with a score “A” (Chipere, 2001). The students were tested on comprehension and recall of complex NP sentences. The results indicated that great differences existed in the comprehension performances with the LAA group performing much worse. Then, the LAA group was divided into two subgroups. One subgroup received memory training and the other group received comprehension training which involved explicit instruction and also the practice session. The results of the new complex NP sentences comprehension showed that memory training led to only improvement in recall task and comprehension training led to improvement on both recall task and comprehension task. The results from this research suggested the important role of input or experience especially on particular grammatical structures.

Similarly, Street and Dąbrowska (2010) conducted two experiments to explore the differences in passive structures and quantifiers between HAA participants and low LAA participants. Experiment 1 demonstrated considerable individual differences in these adult native speakers which were strongly connected with their educational attainments. While the HAA participants performed at ceiling in both conditions, the LAA performed worse. Experiment 2 was conducted with another group of participants. The results showed that training led to significant improvement only on structure trained. This demonstrated that the factor experience or input played an important role in L1 syntactic entrenchment. That is to say, as the HAA group might receive much more input or experience with the syntactic structures than the LAA group, its corresponding representations might be entrenched deeper than those of the LAA group. Thus, the HAA performed better than the LAA in comprehension test.

According to the usage-based models of language (Barlow and Kemmer, 2000; Langacker, 2000; Bybee, 2006), structure emerges from use. In other words, linguistic knowledge or underlying syntax is shaped by usage factors such as input. As input that the language learner is exposed to contains recurrent patterns or means of the repeated structures, then the syntax with the special structures becomes entrenched through repeated use or input. Input together with cognitive factors forms the representation. These models predict frequency effects and individual differences which might be attributable to input factors. In accordance with these models and the studies above, repeated input with specific structures entrenches the representation deeper, and the entrenched syntactic representation brings better performance in language comprehension, etc. Here a meaningful question is raised whether the repeated input or use refers to the identical input or whether it can be extended to similar structures but in different expressions. Can it can be extended to the similar structures not trained in the training phase, that is, can the transfer effect be predicted? What mechanism might facilitate its occurrence? Is there a possibility that it originates from the entrenched representation? It could be interesting to explore the possible connection between the transfer effect and the usage-based theory.

### The Input Factor and L2 Syntactic Processing

In recent years, the body of research dealing with syntactic acquisition of a non-native language has grown greatly, especially the acquisition of late L2 learners. The late L2 learners are those who began L2 learning after a critical period (roughly adolescence) and are thought to encounter great difficulty in L2 acquisition (Foucart and Frenck-Mestre, 2012). Many researchers have shown great interest in the affects of non-native syntactic acquisition (Juffs and Harrington, 1995; Weber-Fox and Neville, 1996; Hahne, 2001; Jiang, 2004, 2007; Cunnings and Clahsen, 2007; Felser and Roberts, 2007; Morgan-Short et al., 2010, 2012a,b; Van Hell and Tokowicz, 2010; Foote, 2011; Roberts and Felser, 2011; White et al., 2012; Bowden et al., 2013; Hopp, 2013a,b, 2015; Deng et al., 2015, 2017; Boxell and Felser, 2017; Cunnings, 2017; Felser and Drummer, 2017). Many factors are thought to be critical in L2 syntactic acquisition such as the age of acquisition (DeKeyser, 2012, 2013), the L1 background (Chen et al., 2007; Sabourin and Stowe, 2008), the language proficiency (Ojima et al., 2005; Steinhauer et al., 2009; White et al., 2012), the input factor and its training method (Ellis, 2002; Dussias and Sagarra, 2007; Ellis and Collins, 2009; Morgan-Short et al., 2012a,b; Unsworth, 2013; Deng et al., 2015, 2017; Deng and Chen, 2019), grammatical integration ability (Hopp, 2013a,^2015^), etc. For example, DeKeyser (2012) claimed that the age of acquisition was the decisive factor in affecting the syntactic acquisition. Steinhauer et al. (2009) proposed the syntactic processing performances of the non-native language relied on proficiency (Steinhauer et al., 2009).

Among all the factors mentioned above, the input factor, which has been investigated recently, was also thought to be an important factor that affected L2 syntactic acquisition (Morgan-Short et al., 2012a,b; Montrul et al., 2013; Deng et al., 2015). Morgan-Short et al. (2012b) adopted an artificial language training paradigm to explore whether the type of language training crucially impacts syntactic acquisition. The participants were randomly divided into implicit training group and explicit training group. The results showed that, at the high proficiency phase after input training, the implicit training group showed native-like processing paradigm with an anterior positivity followed by P600 component, which indicated the important role of the input and input type in syntactic acquisition.

Deng et al. (2015) provided the electrophysiological evidence that input was a very important factor. A pretest-training-posttest paradigm was adopted to investigate the effect of input training. Participants with relatively low proficiency were recruited. After two intensive and specific input training phases with non-local subject-verb agreement structure as “The price of the car was very high,” the P600 component absent in the pretest was elicited in the posttest of the experimental group. For the control group, as they received other structures training, the P600 component was absent from the pretest to posttest when the syntactic structures were violated compared with the correct versions. This may be due to the entrenched representation, which is in accordance with the cognitive grammar theory in L1 which emphasizes the input factor in syntactic representation entrenchment. As the experimental group received the specific non-local subject-verb agreement structure with singular head noun, an interesting and new issue turned up. Can the experimental group who had been trained with the non-local subject-verb agreement structure show sensitivity to the similar forms of subject-verb agreement structure violations? Was the entrenched representation limited to the subject-verb agreement structure with singular head noun trained, or could it be extended to the similar structure in different expression? What would happen if the experimental group encountered the grammatically violated sentence such as “The girls of the family was very beautiful and polite”? The present study aimed to explore this question about the syntactic transfer within L2 context.

Transfer is thought of as a ubiquitous, continuous, systematic use of selected parts of the immense body of prior knowledge, and it means the use of previously acquired knowledge or skills in new learning or problem-solving situations (Steiner, 2001). As the materials of the present study were quite similar to those of Deng et al. (2015), the only differences were the singular or plural head noun and their corresponding predicate. Then, according to the similarity theory provided by Thorndike and Woodworth (1901), the transfer could happen in cases where common elements were shared between the source and the target. Transfer increased proportionally with the number of such overlapping associations in the learning and the test tasks (Thorndike, 1913). This means that the similarity between previous and actual learning content and processes may play a crucial role. This transfer effect, called linguistic transfer, has also been widely studied in the language field (Kellerman, 1979; Sharwood Smith, 1986; Bertram and Kuperman, 2019). Linguistic transfer mainly focused on the cross-language transfer, that is, from L2 to L1 or L1 to L2 on syntactic, lexical, and metalinguistic levels (Kellerman, 1979; Sharwood Smith, 1986; Pavlenko and Jarvis, 2002; Brown and Gullberg, 2008). Syntactic transfer here refers to the ability of using the entrenched representation in processing the new syntactic structure that is not trained. Yet, little attention is paid to the intralinguistic transfer effect within the L2 context which is of great importance. The present study set to explore this syntactic transfer effect by focusing on the subject-verb agreement structure. As the materials trained and investigated highly overlap, the transfer effect might take place.

### The Current Study

The current study aimed to investigate transfer effect of linguistic input training in late L1-Chinese L2-English learners within the L2 context. The participants who took part in the study of Deng et al. (2015) were invited back to participate in this independent transfer effect test.

As introduced above, in Deng and colleagues’ studies, the materials for the training phases were non-local subject-verb agreement structure with singular head noun, while the similar structure with plural head noun was not provided in that study. Though the previous study provides evidence for the relationship between input and representation entrenchment, it is only limited to a specific structure, that is, the structure with a singular head noun. As the routine paradigm of the training-related studies, transfer effect thus comes into consideration. The present study aimed to explore the transfer effect for the similar structure with a plural head noun. By exploring this syntactic transfer effect, we tried to answer the research questions as follows: Could the relationship between the input and syntactic representation be one-to-one correspondence? Or could it be extended to the broader syntactic category? Could these late L2 learners still show plasticity in L2 syntactic acquisition?

As the Chinese language is well known for its impoverished system of grammatical morphology (Li et al., 1993, 2004) and Chinese syntax does not require any subject-verb agreement (Li et al., 1993; Jiang, 2004; Chen et al., 2007) and any nominal subject (Chen et al., 2007), it was difficult for Chinese learners to successfully acquire this structure. The subject-verb agreement structure in English includes several expressions as local “The boy usually goes to school by bus,” non-local as “The price of the cars was very high,” etc. Though the experimental group in the study of Deng et al. (2015) showed sensitivity to the syntactic violations after two sessions of training, it is still unknown whether this specific input training with singular head noun could be transferred to the same subject-verb agreement structure but in different expressions. Therefore, the present study aimed to take a small step in exploring transfer effect in late English learners. Specifically, we are concerned with whether participants showed sensitivity to the subject-verb agreement violations with plural head nouns which were not trained.

In this transfer effect test, the experimental materials were sentences with violations of non-local subject-verb agreement with plural head nouns and their grammatical counterparts. These sentences were newly constructed for the transfer effect experiment.

To sum up, the present study used the ERP technique to investigate the transfer effect of linguistic input training in late Chinese-English learners, using subject-verb agreement structures with plural head nouns as the stimuli. The focus will be on the P600 component as the indicator.

## Materials and Methods

### Materials

The materials in this transfer experiment were all newly constructed. The experiment consisted of the non-local subject-verb agreement structure with plural head nouns, but it differs from the materials in the study of Deng et al. (2015).

The materials in this transfer experiment were non-local subject-verb agreement with plural head nouns, including the grammatical correct sentences and the grammatical incorrect counterpart. After the plausibility ratings, 80 pairs of sentences (160 in total) were chosen for the formal transfer experiment: half grammatical and half ungrammatical. To balance the materials, two lists were finally adopted with each list containing 80 experimental sentences (40 correct and 40 incorrect) and 160 fillers in other syntactic structures except subject-verb agreement were included.

The examples of the experimental stimuli are presented in Table 1.

**TABLE 1:** Sentence examples for the transfer experiment.

Group	Sentence type	Examples
EG	PSP_G	The girls of the family were very beautiful.
	PSS_UG	The girls of the family was very beautiful.
	Fillers	She used to eat apples after supper.
CG		This part was the same as the EG.

*EG, experimental group; CG, control group.*

For the abbreviations in the table, the first capital letter “P” represents “plural head noun.” The second capital letter “S” represents “singular local noun.” The third capital letter “P” or “S” represents “plural verb (were)” or “singular verb (was)” respectively. “G” or “UG” means “grammatical” or “ungrammatical.” It should be noted that the sentence marked as PSS-UG is the ungrammatical version of the sentence PSP-G.

Eighteen Chinese college students from the same background as the participants were asked to rate the plausibility of syntactically correct experimental sentences on a 5-point scale where 1 signified *definitely implausible* and 5 signified *perfectly plausible*.

These participants never participated in our formal experiments. Sentences with a mean semantic plausibility above 4 were selected. In total, 80 plausible sentences were chosen for the final transfer experiment (*M*_PSP_ = 4.29, SD = 0.21) on the plausibility rating.

### Participants

The participants, who took part in the experiment of Deng et al. (2015), were invited to come back to participate in this independent transfer effect experiment. Because some participants graduated from school, eventually 26 college students participated in this experiment: 13 participants in the EG (8 females, average age = 23.71 years old, average age of classroom exposure = 12.07 years) and 13 participants in the CG (6 females, average age = 21.21 years old, average age of classroom exposure = 12.21 years). In short, 26 participants participated in this transfer effect test.

All participants were late L1-Chinese L2-English learners. They received classroom teaching of English in China and none had experience of living in English-speaking countries. They had passed CET (College English Test) band 4 but not band 6. CET is a large-scale standardized exam conducted to evaluate the college students’ English proficiency, with 710 as full marks and 425 as the passing line. Band 6 represents a higher proficiency level than Band 4. The test consists of five parts including listening comprehension, reading comprehension, vocabulary knowledge, grammar knowledge, and writings. Only those who have successfully passed the Band 4 were qualified to take the Band 6 exam.

These participants completed the Oxford Placement Test (OPT) and a self-rating questionnaire. The OPT is a standardized objective test for university English foreign language classes, which is proved to be an effective instrument and a reliable means of grading students at all levels (Moll, 1999). It includes 25 multiple-choice questions and a cloze test, and the full mark is 50. The higher the score is, the higher the proficiency is. The 5-point self-rating questionnaire where “1” signifies *quite poor* and “5” *highly proficient* is used to evaluate the participants’ listening, speaking, reading, and writing skills. It is a subjective indicator of English proficiency.

All participants reported being right-handed and having normal or corrected-to-normal vision. A written informed consent form was signed before the formal experiment, and money compensation was provided for the participation.

### Experimental Procedure

The participants who finished the sessions of the study of Deng et al. (2015) were asked to take part in this independent study on transfer effect.

The test sentences were those that were non-local subject-verb agreement structure with plural head nouns. Two counterbalanced lists were created. Participants were randomly distributed to one of the two lists. One hundred and sixty fillers were constructed with half of the fillers being syntactically incorrect (i.e., containing violations in verb subcategorizations or reflexive pronouns, etc., for example, the violation in reflexive pronoun of the sentence “The boy quickly adopt herself to new circumstances”) and the other half being simple grammatical sentences with various sentence structures.

Participants’ ERPs were recorded when they were reading the test sentences. In accordance with the previous study (Havas et al., 2012), each trial began with an asterisk fixation (500 ms) in the center of the screen, followed by test sentences that were presented word-by-word in the center of the screen (500 ms per word with an inter-stimulus interval of 500 ms). The last word of each sentence was followed by two asterisks indicating the end of the sentence. Half of the sentences were followed by a comprehension question to make sure the participants read the sentences attentively. The comprehension questions were relatively easy in order to not cause much pressure for participants. For example, the question for the example (1) listed above was “Were the girls very beautiful?” Participants were tested individually in a quiet room and were asked to minimize their blinks and body movements. The ERP recording session began with 10 practice trials to make sure the participants were familiar with the procedure. They could take a short break every 40 trials. Each ERP recording session lasted about 2 h including preparations.

### Event-Related Potentials Data Analysis

EEG signals were recorded at a 1,000 Hz sampling rate from a 64-channel Quik-cap with Ag/AgCI electrodes. EEG electrodes were placed according to the extended 10–20 system. All electrodes were referenced to the left mastoid during recording and off-line referenced to linked mastoids. Impedances were kept below 5 KΩ. Eye movements were measured using vertical EOG with two electrodes placed above and below the left eyes, and the horizontal EOG with two electrodes placed to the outer canthi of the two eyes. EEG data analysis was performed using Scan 4.3. The electrophysiological signals were filtered with a bandpass of 0.05–100 Hz (half-amplitude cutoffs) at a sampling rate of 500 Hz. In the off-line analysis, the EEG was filtered with a 0.12–40 Hz band-pass filter. Trials with voltage exceeding ± 90 μV and trials with eye movements were excluded from ERP averages, resulting in an exclusion of about 12.6% and 10.4% of the trials in the EG and the CG respectively. As the task used in the present study was sentence comprehension, both trials answered correctly and incorrectly were included in the EEG data analysis. ERPs time-locked to the onset of the violation word (i.e., “was”) or matched control word (i.e., “were”) were averaged for each participant for all the electrodes from −200 to 1,000 ms.

The main ERP component of interest was P600, which is maximal at centro-parietal electrodes (Bowden et al., 2013). Therefore, the electrodes selected for data analysis after visual inspection were: left region (C5, CP5, P5, PO5), central region (Cz, CPz, Pz, POz), right region (C6, CP6, P6, PO6). Time window of 500–1,000 ms was selected to analyze P600 in accordance with the previous research (Chen et al., 2007).

Mean amplitudes for each time window were analyzed using a global ANOVA with the between-subject factor Group (experimental, control), and the within-subject factors Grammatical condition (grammatical, ungrammatical), and Laterality (left, central, right). For convenience, we used the abbreviation Gro instead of Group, Gra as the abbreviation of Grammaticality, and L as the abbreviation of Laterality in the following content. Significance levels of the F ratios were adjusted with Greenhouse-Geisser correction. As we only cared about whether participants in the experimental group could elicit the P600 component, any global ANOVA that yielded any significant (*p* < 0.05) interaction involving the factor Grammatical condition and Group was followed up with the step-down ANOVAs to clarify the nature of the interaction.

## Results

### Oxford Placement Test Results and Self-Rating Results

Mean age of first English classroom exposure, English self-rating scores, and OPT scores are presented in Table 2. The *t*-test results showed that there were no significant differences between the two groups in any of the proficiency measures (*p*s > 0.05), indicating that they were well matched.

**TABLE 2:** Means age of first English classroom exposure (years), English self-ratings and OPT scores (standard deviations in parentheses).

Group	EG	CG
Age of first English classroom exposure	12.07 (0.83)	12.21 (1.71)
Listening	3.07 (1.14)	2.85 (1.09)
Speaking	3.07 (0.62)	2.85 (0.66)
Reading	2.93 (1.07)	2.57 (0.65)
Writing	2.61 (1.56)	2.57 (1.08)
OPT	38.04 (3.53)	38.00 (2.89)

*EG, experimental group; CG, control group; OPT, Oxford Placement Test.*

### Behavioral Results

The behavioral accuracy of the two groups in this transfer effect test is presented in Table 3.

**TABLE 3:** Mean accuracy (%) and standard deviations (SD) for sentence comprehension.

Group	Accuracy	SD
Experimental group	83.78	3.97
Control group	83.70	2.91

Paired-samples *t*-test showed that there was no significant difference between the two groups (*p* > 0.1), indicating that they did not differ in sentence comprehension.

### Event-Related Potentials Results

The global ANOVAs including the between-subject factor Group (experimental, control), the within-subjects factors Grammatical condition (grammatical, ungrammatical), and Laterality (left, central, right) was conducted, respectively, in the 300–500 ms and 500–1,000 ms time window. Results are listed in Table 4.

**TABLE 4:** Summary of global ANOVA for the two groups.

Source	Df	*F* value	
		300–500 ms	500–1,000 ms
Gra	1.24	4.23	3.65
Gra × Gro	1.24	3.88	5.00*
Gra × Gro × L	2.48	1.12	0.49
Gra × L	2.48	1.43	0.04

*Gra, grammaticality; Gro, group; L, laterality.*

*p < 0.05.

### 300–500 Time Window

Results in this time window showed that the main effects and the interactions were not significant (*ps* > 0.05).

### 500–1,000 Time Window

In the time window of 500–1,000 ms, results of the global ANOVA showed that the interaction between Grammatical condition and Group [*F*(1, 24) = 5.00, *p* = 0.035, η*_p_^2^* = 0.17] was significant. Other main effects and interactions were not significant (*ps* > 0.05). In order to clarify the nature of the interaction between Group and Grammatical condition, further analysis by Group was conducted.

**FIGURE 1:**
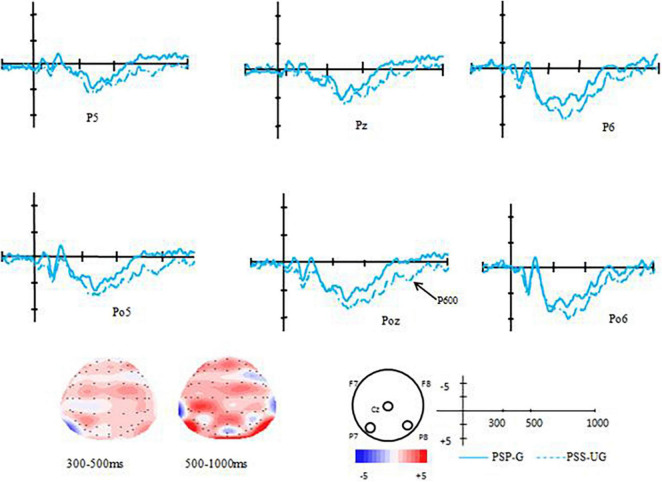
Voltage maps and the grand average ERPs of the EG for the difference between correct and violated sentences in the transfer-effect test.

**FIGURE 2:**
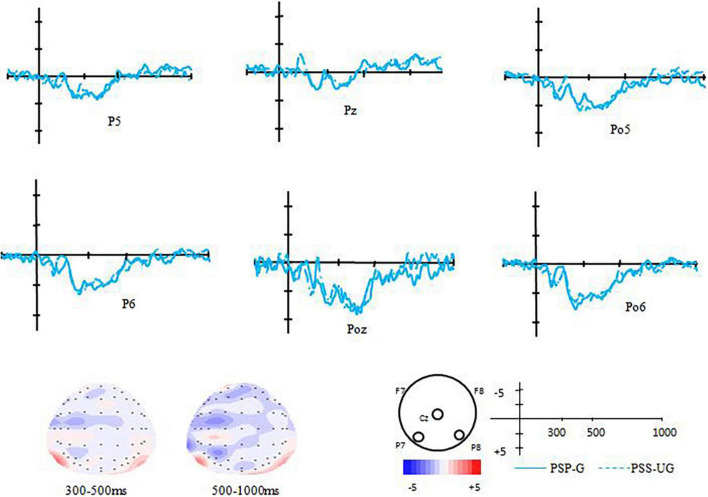
Voltage maps and the grand average ERPs of the CG for the difference between correct and violated sentences in the transfer-effect test.

For the experimental group (EG), the results of the paired-samples *t*-test between the grammatical condition and ungrammatical condition showed that difference between these two conditions was significant [*t*(12) = 2.814, *p* = 0.016, *d* = 0.85], that was the ungrammatical condition elicited a more positive component than that of the grammatical condition (0.46 μV vs. −0.66 μV). According to its distribution, it should be termed as P600 (see Figure 1), indicating obvious transfer effect. However, for the control group (CG), the results of the paired-samples *t*-test revealed no significant difference between the grammatical condition and the ungrammatical condition (*ps* > 0.05) (see Figure 2).

## Discussion

The present study investigated the transfer effect in late L2 learners to explore whether the relationship between input and the entrenched representation was extended to the broader category of the subject-verb agreement structure, and whether these late L2 learners still show plasticity in syntactic acquisition. Results showed that syntactic violations which were evident in the subject-verb agreement structure with plural head nouns elicited a significant P600 in the experimental group (EG) but not in the control group (CG). The findings indicated a significant syntactic transfer effect, which indicated the important role of input in L2 syntactic acquisition of the late L2 learners.

### The Role of Input, L2 Syntactic Transfer, and Late L2 Learners

The experimental group indicated a significant P600 to the violations of the subject-verb agreement structure with plural nouns, compared with the grammatical counterpart, while the control group did not. The P600, as the index of grammaticalization (White et al., 2012) and indicator of entrenched representation (Deng et al., 2015), can be regarded as the instantiation of grammatical knowledge into the learners’ online language processing system (Osterhout et al., 2008) and increased accessibility originated from the entrenched representation (Bybee, 2006). Participants from the EG benefited from the specific trainings with subject-verb agreement structures with singular head nouns and had their corresponding representation entrenched, which might give rise to the elicitation of P600. The results were in accordance with the related studies on input in L2 field (Morgan-Short et al., 2012a,b; Montrul et al., 2013; Deng et al., 2015). Additionally, the neural processes underlying L2 (morpho) syntax especially for late L2 learners are thought to be complicated. Some studies showed the biphasic components of LAN and P600 (Deutsch and Bentin, 2001; Friederici et al., 2002; Hahne et al., 2006; Steinhauer et al., 2009; Zawiszewski and Friederici, 2009), while others showed only the P600 (Bowden et al., 2007). The only P600 component, without the LANs component, might indicate that the EG participants were not proficient enough with the subject-verb agreement structure to reach the automatic processing, according to White et al. (2012).

Due to insufficient input with the specific subject-verb agreement structure, participants of both the EG and CG showed no sensibility to violations of this structure in the pretest of Deng et al. (2015). Two intensive training sessions with specific subject-verb agreement structures made participants in the experimental group relatively proficient with this specific structure (White et al., 2012) and thus made its corresponding representations entrenched (Bybee, 2006). Though the participants were not provided with the subject-verb agreement structures with plural head nouns in the training sessions (Deng et al., 2015), the EG still showed sensitivity to these violations, that is, syntactic transfer effect, which might be due to the entrenched representation. The P600 component of this syntactic transfer effect might be the indication of the role of input not only in entrenching the corresponding the one-to-one corresponding representation (Deng et al., 2015; Deng and Chen, 2019) but also in entrenching the broader category of the syntactic representation.

Questions remains in the L2 field regarding what might affect the L2 syntactic acquisition: the AoA (age of acquisition, thus the distinction the early learners and late learners), the level of proficiency, input and other variables as learning strategy, etc. (Lenneberg, 1967; De Haene et al., 1997; Chee et al., 2001; Reiterer et al., 2005, 2009; White et al., 2012). Some researchers suggested that early learners outperformed late learners due to the age of onset (Lenneberg, 1967; De Haene et al., 1997). Some insisted that it might be the proficiency level that decided the acquisitions (Steinhauer et al., 2009). The fact that the participants of the present study who were later learners with relatively low general proficiency showed a significant transfer effect cannot be simply attributed to the factor of AOA or the general proficiency level. The relationship between input and transfer effect of the present study might partly indicate that input might be an important factor behind AOA and proficiency. Distinction between early and late leaners lies not only in age but also the possible input or exposure. Similarly, proficiency is the outcome variable, as claimed by Reiterer et al. (2009), and it is a complex variable that functions as an umbrella term and subsumes many of the other factors such as input training (Reiterer et al., 2009). Input training might improve proficiency level with the specific structure trained, which is thought to be the possible important factor affecting the processing performance (White et al., 2012; Deng et al., 2015). Though the present study, together with other studies (Morgan-Short et al., 2012a,b; Montrul et al., 2013; Deng et al., 2015), explored the role of input in L2, it still calls for more efforts in this L2 field.

Previous studies exploring the transfer effect mainly focused on the inter-language influence, that is, from L1 to L2 or L2 to L1 to explore the role of background language or the directionality (Kellerman, 1979; Sharwood Smith, 1986; Pavlenko and Jarvis, 2002; Brown and Gullberg, 2008), while very few studies focused on the intralinguistic syntactic transfer effect. The elicitation of P600 in EG, as the indicator of the syntactic transfer effect, not only indicated the role of input in intralinguistic syntactic transfer effect but also made a relatively small step in extending the studies on the role of input in L2.

In short, these late L2 learners of the present study, who indicated syntactic transfer effect, might give some enlightenment in the L2 field: First, input factor plays an important role in L2 one-to-one syntactic entrenchment and also syntactic transfer effect, which is in accordance with the studies both in L1 and L2 (Kaschak and Glenberg, 2004; Bybee, 2006; Dabrowska, 2008a,b; Street and Dąbrowska, 2010; Morgan-Short et al., 2012a,b). Even for late L2 learners, input training still matters. Second, the variables in the L2 syntactic field are very complicated and many factors intertwine with each other. Maybe in the future experimental techniques could be used to disentangle the complicated relationships among the variables to acquire relatively pure results.

### The Transfer Effect and Its Probable Occurrence Mechanism

In Deng et al. (2015), no ERP components were observed in either EG or GG upon syntactic violations in the pre-test. Then a significant difference was observed between the EG and the CG in an immediately post-test after input training, with a P600 elicited in the EG but not in the CG. This revealed the importance of input training in L2 representation entrenchment, and also that the entrenched representation effect can last a relatively long time (Deng and Chen, 2019). However, questions still remain: Is the entrenched representation only limited to the structure trained, or can it be transferred to the similar structure? What might give an explanation for this transfer effect?

In the present transfer study, a significant P600 was still elicited in the EG but not in the CG, indicating the obvious transfer effect to the subject-verb agreement with plural head nouns. We attempt to explain our results within usage-based theory and similarity theory.

According to usage-based theory, input or usage strengthens the memory representations, making them easier to access (Goldberg, 1995; Croft, 2000; Bybee, 2006; Schmid, 2007). Every exemplar of a language use or input encountered by a speaker has an effect on cognitive representation in memory. As claimed by Bybee (2006), the linguistic memories represented as exemplars can undergo considerable reorganization. Exemplars of phrases or sentences that are similar on different dimensions are grouped together in cognitive representation. Similar but not identical exemplars are stored and represented to constitute a cluster or categories. Eventually, as long as the cognitive representation has been entrenched to a certain degree, it turns out to be highly effective, accessible, and autonomous (Goldberg, 1995; Croft, 2000; Bybee, 2006). Increase in input or exposure leads to representation entrenchment, accessibility of a preexisting representation, learning or acquisition of a new representation, or reorganization or modifications to existing representations (Luka and Choi, 2012). Thus, speakers might improve their performance in comprehending such exemplar categories or cluster based on the entrenched representation, which might be in accord with the previous studies in that appropriate representations increase positive transfer (Luchins, 1942; Chen and Daehler, 1989; Singley and Anderson, 1989).

As to the participants here, although they belong to the participants with relatively low proficiency, they have systematically learned the grammar about the subject-verb agreement structures explicitly in classroom teaching. This kind of input is not sufficient enough for them to form deep representation, which is evident in the lack of P600 in the pretest in the study of Deng et al. (2015). Then, the EG received the specific input training on the subject-verb agreement structure with singular head nouns, which entrenched their relatively shallow representation. The P600 of the EG in the posttest provided the evidence. This kind of non-local subject-verb agreement structure with singular head noun provided in the training sessions, and the materials about the non-local subject-verb agreement structure with plural head noun, belong to this same grammatical category or cluster. According to the usage-based theory (Goldberg, 1995; Croft, 2000; Langacker, 2000; Bybee, 2006; Haskell et al., 2010), exemplars provided in the input training sessions, together with the previous subject-verb agreement structures that the participants learned in the classroom, are grouped together in cognitive representation, where linguistic memories represented as exemplars can undergo considerable reorganization or reanalysis. That is, the syntactic representation about subject-verb agreement expressed in similar or different exemplars together might have been entrenched, through the two sessions of input training provided (Goldberg, 1995; Langacker, 2000; Bybee, 2006). Then, the entrenched representation gave rise to autonomy and efficacy in comprehending the subject-verb agreement structure with plural head nouns, where the P600 to the agreement violations in the EG might provide evidence.

The second explanation for this occurrence mechanism of the transfer effect might be the high similarity between the materials tested in the present study and those trained in the study of Deng et al. (2015). According to Thorndike’ s classical view on the transfer effect (Thorndike and Woodworth, 1901), the likelihood of the occurrence of the transfer effect is directly related to the similarity between the situations trained and the situations tested. As claimed by Thorndike (1913), transfer could take place in cases where common elements were shared between the source and the target. Specifically, the materials’ structure of the present study contained a plural countable noun modified by a prepositional phrase (PP), the verb “were” or “was” as the predicate that either agreed or disagreed with the plural subject noun in number, and other sentence constituents. For example, “The girls of the family were (was) very beautiful and polite.” That structure of Deng et al. (2015) included a single countable noun modified by a prepositional phrase (PP), the verb “was” or “were” as the predicate that either agreed or disagreed with the singular subject noun in number, and other sentence constituents. For example, “The price of the car was (were) very high.” They were quite similar in structure expressions. Then, according to the similarity theory, the greater the similarity between the learning task and the test task, the higher the possibility that the transfer could take place (Thorndike, 1913). The only difference between the materials of the present study and the previous study of Deng et al. (2015) was whether the head noun was plural or singular. Therefore, it might be impossible to tease apart the effect of the similarity between the materials on the transfer performance.

In brief, the present study not only provided the evidence for the transfer effect but also tried to give possible explanation about how this transfer effect happened both from the perspective of the usage-based theory (Bybee, 2006) and the similarity theory (Thorndike and Woodworth, 1901). Also, it will be interesting to design some experiments to explore what decides the occurrence of the transfer effect in the future.

### The Essence of the L2 Grammar System and Its Broad Significance

In L2 syntactic acquisition, the question of whether the current attainment state of the late L2 learners is the ultimate attainment is still open, as late L2 learners began their L2 learning late in life. This question concerns the possibility for development of late L2 learners. One of the prevalent opinions is that maturational changes lead to discontinuity in the neurocognitive architecture in language development. As a consequence, native-like outcomes in language acquisition are argued to be limited in a biologically circumscribed period of time when language acquisition needs to begin, usually taken to end in late childhood or around puberty (Singleton and Ryan, 2004; DeKeyser, 2012, 2013). That is to say, according to this critical period hypothesis, it might be the ultimate attainment or the end-state of the late L2 learners who began to learn the L2 after puberty, indicating that L2 syntax of the late L2 learners is considered to have a developmental endpoint. Some studies indicated that even advanced late L2 learners occasionally showed slip-ups or even protracted variability in subject-verb agreement, tense, and gender marking (Jiang, 2004, 2007; Silva and Clahsen, 2008), which might be attributed to age constraints. In contrast to this opinion, some researchers believe that it is the proficiency or input factor that affects the acquisition of the L2. As the proficiency or input improves, their difficulty in syntactic comprehension can be relieved (Steinhauer et al., 2009; White et al., 2012). For instance, Deng et al. (2015) conducted an experiment to explore the role of the input in L2 syntactic processing (Deng et al., 2015). The results indicated that the input plays an important role in improving structure-specific proficiency and entrenching syntactic representation. And in Deng and Chen (2019), the results showed that the entrenched representation can even be maintained in a relatively long period. Both of these studies, coupled with the transfer effect of the present study, show the plasticity and dynamic nature of the L2 grammar system. Even for late learners, their grammar system can be in constant grammaticalization of syntactic rules through language exposure.

The transfer effect reported in the present study provides evidence for the malleability of late L2 learners’ grammatical system, which is in line with the results of L1 studies (Kaschak and Glenberg, 2004; Kaschak, 2006; Kaschak and Borreggine, 2008; Wells et al., 2009; Haskell et al., 2010; Kaschak et al., 2011; Luka and Choi, 2012). The malleability of the adult language in the aspect of syntax development is robustly evident in a set of phenomena broadly called structural priming, which shows that exposure to a given syntactic construction can affect the subsequent processing of the same or related constructions (Bock, 1986; Branigan et al., 1999, 2000; Pickering and Ferreira, 2008). According to these theories, learning should be reserved for more enduring changes. These observations suggest that incremental adjustments to the language processing system occur continuously and may even extend to acquisition of novel syntactic structures. For instance, Kaschak and Glenberg (2004) carried out a series of experiments to explore how adults learned to comprehend a new syntactic construction in their native language. In experiments 1 and 2, the adults quickly learned to comprehend the new “need” construction and generalized it to new verbs. The transfer effect was evident in which the participants learned to comprehend a novel syntactic pattern from only a few exposures buried within about 10–12 min of conversation. Participants who learned to comprehend the “need” construction were able to generalize this learning to processing the same construction with a new verb. The results indicated that the mechanism that functions in child language acquisition may play an important role in adults’ continued ability to learn new constructions in their native language (Seidenberg and MacDonald, 1999). In other words, for adults, syntactic learning is a continuous, dynamic process throughout the whole life span. The results of the present study are consistent with that of Kaschak and Glenberg (2004). According to our results, for late L2 learners, the current state of the grammatical knowledge in memory is not the end-state of the attainment. Their syntactic representation can be entrenched dynamically with the joint forces of input or exposure, cognition, and their interaction.

In short, the transfer effect in the present study indicated that even for late L2 learners, the malleability and learnability of syntax is possible. The nature of grammatical knowledge in memory is dynamic. For late L2 learners, their learning grammatical and morphological knowledge can bring changes and might continue throughout the lifetime.

What’s more, the results of the transfer effect might provide some pedagogical implications as to the training paradigm. First, intensive training with specific syntactic structure is like a structure-oriented approach that can contribute to proceduralizing the known syntactic rules. This kind of approach might help participants rediscover already known language in their direct contact with new content input. Second, the training method, the self-paced reading, feedback-facilitated method, not only closely resembled natural reading but also involves a higher level of linguistic and cognitive processes such as inference-making (Just et al., 1982), as the participants have to summon their existing syntactic knowledge and reading strategies to correctly comprehend the sentences in this moving window condition. Third, this kind of task focuses the participants’ attention on each word they read, which might indicate the important role of attention in L2 syntactic acquisition. As claimed by Devos (2016), “to encourage FL practice and simultaneously mitigate fossilization, specific attention needs to be paid to the language. The self-paced reading paradigm,” the structure-oriented approach with specific subject-verb agreement structures, together with the grammatical judgment task after reading, specifically directed the participants’ attention to the head noun and its corresponding predicate. Maybe, in the future, when the teachers design the tasks to help students acquire the syntax, they might take into the consideration such elements as cognition, attention, etc.

Nevertheless, there are important limitations to the present study. First, our studies concerned the transfer effect of the same subject-verb agreement structure with the only difference in head noun. To gain a broader picture of the late L2 learners’ ability in syntactic acquisition, similar studies should be done with a range of other transfer conditions. For example, could the participants show sensitivity to other subject-verb agreement expressions after being trained with the materials in the present study? Or was the transfer effect specific to this experimental context? Without a definitive answer, the conclusion that can be drawn from these data is that training with specific structures can lead to easier comprehension of the similar structure. Second, because there are high-level similarities between the trained materials and those of the present study, it might be difficult to differentiate what leads to the present results. Is the transfer effect attributed to similarity or to entrenched representation or to both?

The present experiment takes an important step toward understanding how late L2 learners learn to process similar structure. Of course, our results might only scratch the surface about the interaction between learning, memory, representation, language acquisition, and language processing. More research is needed to shed light on these interactions with the late L2 learners to clarify the internal language acquisition mechanism. For example, it might be interesting and meaningful to explore, how long does this kind of syntactic transfer effect last and what factors are likely to affect the sustainability? The present study makes an effort to shed some light on the complex L2 syntax acquisition abilities of late L2 learners.

## Conclusion

In conclusion, the present study set to explore such questions as: Could the relationship between the input and syntactic representation be one-to-one correspondence? Or could it be extended to the broader syntactic category? Could these late L2 learners still show plasticity in L2 syntactic acquisition? The current results of the present study suggest that linguistic input training contributes to the transfer effect, indicating the malleability and dynamic nature of the L2 syntactic acquisition. Input plays an important role not only in one-to-one L2 syntactic representation entrenchment but also in the entrenchment of a broader category of the syntactic representation. Last but not least, even for the late L2 learners, learning and plasticity in L2 syntactic aspect can continue. The results of the present study provide not only theoretical implications on the learnability of late L2 learners but also the pedagogical implications for the teachers on the task designs.

## Data Availability Statement

The raw data supporting the conclusions of this article will be made available by the authors, without undue reservation.

## Ethics Statement

The studies involving human participants were reviewed and approved by Beijing Normal university. The patients/participants provided their written informed consent to participate in this study.

## Author Contributions

TD and QF contributed to the design and manuscript drafting. DD provided help in data collecting and processing. All authors contributed to the article and approved the submitted version.

## Conflict of Interest

The authors declare that the research was conducted in the absence of any commercial or financial relationships that could be construed as a potential conflict of interest.

## Publisher’s Note

All claims expressed in this article are solely those of the authors and do not necessarily represent those of their affiliated organizations, or those of the publisher, the editors and the reviewers. Any product that may be evaluated in this article, or claim that may be made by its manufacturer, is not guaranteed or endorsed by the publisher.
